# ^11^C-Labeling of Aryl Ketones as Candidate Histamine Subtype-3 Receptor PET Radioligands through Pd(0)-Mediated ^11^C-Carbonylative Coupling

**DOI:** 10.3390/molecules22050792

**Published:** 2017-05-12

**Authors:** Fabrice G. Siméon, William J. Culligan, Shuiyu Lu, Victor W. Pike

**Affiliations:** Molecular Imaging Branch, National Institute of Mental Health, National Institutes of Health, Bethesda, MD 20892-1003, USA; simeonf@mail.nih.gov (F.G.S.); wjculligan@gmail.com (W.J.C.); shuiyu.lu@mail.nih.gov (S.L.)

**Keywords:** [^11^C]carbon monoxide, histamine 3 subtype, carbon-11, carbonylative coupling, radioligand

## Abstract

Pd(0)-mediated coupling between iodoarenes, [^11^C]carbon monoxide and aryltributylstannanes has been used to prepare simple model [^11^C]aryl ketones. Here, we aimed to label four 2-aminoethylbenzofuran chemotype based molecules ([^11^C]**1**–**4**) in the carbonyl position, as prospective positron emission tomography (PET) radioligands for the histamine subtype 3 receptor (H3R) by adapting this methodology with use of aryltrimethylstannanes. Radiosynthesis was successfully performed on a platform equipped with a mini-autoclave and a liquid handling robotic arm, within a lead-shielded hot-cell. Candidate radioligands were readily formulated in saline containing ethanol (10%, *v*/*v*) and ascorbic acid (0.5 mg/10 mL). Yields for preclinical use were in the range of 5–9%, decay-corrected from cyclotron-produced [^11^C]CO_2_ and molar activities were >115 GBq/µmol at end of synthesis. Radiochemical purities exceeded >97%.

## 1. Introduction

Histamine subtype-3 receptors (H3Rs) are located on presynaptic nerve terminals where they regulate the synthesis and release of histamine and also modulate the release of other neurotransmitters, notably dopamine, serotonin, norepinephrine and γ-aminobutyric acid [1,2]. H3R inverse agonists have proven clinical efficacy for the treatment of neuropsychiatric and neurodegenerative disorders, such as excessive diurnal sleepiness in patients with narcolepsy or Parkinson’s disease, Alzheimer’s disease, schizophrenia, narcolepsy and attention deficit disorders [3,4]. H3R inverse agonists are also a source of leads for developing positron emission tomography (PET) radioligands for H3R, which could be powerful tools for (i) elucidating differences in H3R distribution and density between healthy and disease states; and (ii) for determining the dose-dependence and duration of brain H3R receptor occupancy by drug candidates.

Several ^18^F (*t*_1/2_ = 109.8 min) and ^11^C (*t*_1/2_ = 20.4 min) labeled H3R radioligands have emerged based on imidazole and on non-imidazole chemotypes [5,6,7,8,9]. The non-imidazole chemotype has provided radioligands with imaging performance in non-human primates and human subjects superior to that of the imidazole chemotype. In our continuing efforts to develop more selective PET radioligands for H3R based on a 2-aminoethylbenzofuran chemotype, we recently reported the 2-aminoethylbenzofuran derivative [^18^F]**2** as a putative high-affinity radioligand to image H3R in vivo with PET and discovered that the nitro-precursor (**1**) of [^18^F]**2** was also a selective and high-affinity H3R ligand [7]. We hypothesized that **1** could be labeled with carbon-11 and that similar labeling of potent congeners of **2** might provide alternative radioligands with more choice of starting material, labeling site, and metabolic profile. An added advantage of a carbon-11 label over a fluorine-18 label is that it allows two PET scans in one day in the same subject.

**Scheme 1:**
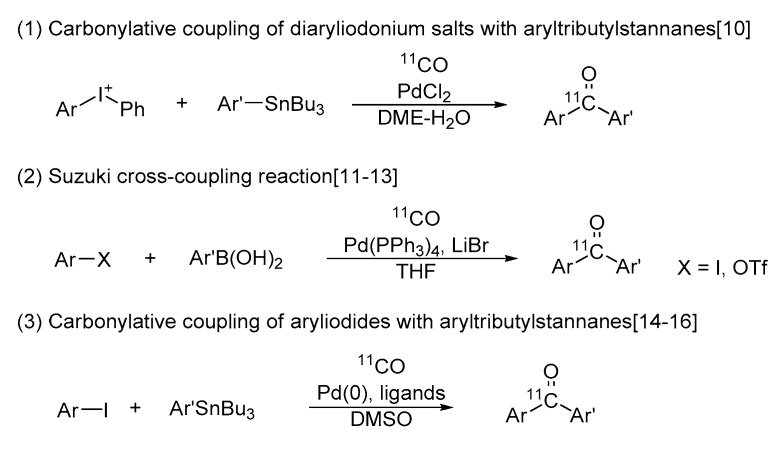
Methods for synthesis of [^11^C]aryl ketones.

[^11^C]Aryl ketones have been prepared by Pd(0)-mediated [^11^C]carbonylative coupling reactions of diaryliodonium salts with aryltributylstannanes [10], and iodoarenes or aryl triflates with boronic acids (Suzuki reactions) [11,12,13] or aryltrialkylstannanes (Stille reactions) [14,15,16] (Scheme 1). The reagents used in these methods are not overly sensitive to atmospheric oxygen and moisture. These methods complement each other and widen the choice of synthetic strategies and starting materials. Nevertheless, up to now, literature examples exploring methodology development have been largely limited to the labeling of simple substrates with limited structural complexity and functionality. Only one example of a PET radioligand labeled with carbon-11 in a ketone carbonyl position has been reported in the open literature [17]. Here, we aimed to synthesize four candidate H3R radioligands ([^11^C]**1**–**4**) by reaction of a common iodoarene precursor (**11**) with one of four aryltrimethylstannyl derivatives (**12**–**15**) via Pd(0)-mediated [^11^C]CO coupling.

## 2. Results and Discussion

### 2.1. Lead Compound Selection and Synthesis of Non-Radioactive Standards

We initially selected **1**–**4** (Table 1) as lead compounds based on their reported high affinities for human H3R and some reported attractive pharmacokinetic properties (e.g., good brain penetration, adequate half-life in vivo) [18], plus moderate computed lipophilicities (cLogD values), all of which were considered promising for PET radioligand development [19,20].

**Scheme 2:**
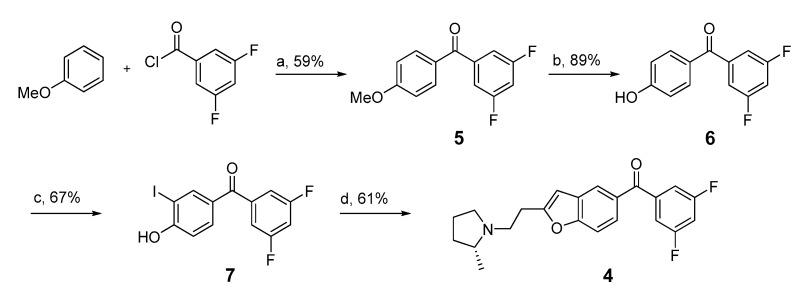
New synthetic route for compound **4**. *Conditions*: (**a**) Sc(OTf)_3_; (**b**) BBr_3_, −78 °C, DCM; (**c**) KI, I_2_, NH_4_OH; (**d**) (*R*)-1-(but-3-ynyl)-2-methylpyrrolidine in MeCN, Pd(PPh_3_)_2_Cl_2_, CuI, NEt_3_, KI, DMF.

Compounds **1**–**3** were prepared as reported previously [18] to provide reference materials and samples for screening in pharmacological binding assays. The preparation of **4** was described previously as a 6-step synthesis, starting with 4-benzyloxybenzoic acid, in low overall yield (<6%). Instead of using this procedure, we designed and implemented a simplified 4-step procedure starting with 3,5-difluorobenzoyl chloride that afforded **4** in an improved 22% overall yield (Scheme 2). First, 3,5-difluorobenzoyl chloride was treated with anisole in the presence of scandium triflate to give **5** (59%). The **5** was demethylated with boron tribromide to give **6** (89%). The resultant phenol was treated with iodine and potassium iodide in basic solution to give **7** (67%). Coupling of **7** with (*R*)-1-(but-3-ynyl)-2-methylpyrrolidine gave the target compound **4** in 61% yield.

In our evaluation, all four ligands exhibited high human H_3_ receptor affinity and high selectivity (>200 fold) over other histamine receptor subtypes (Table 1), thereby affirming their suitabilities in these regards for consideration as potential candidate PET radioligands.

### 2.2. Synthesis of Iodo-Precursor *(**11**)* and Stannyl Derivatives *(**12**–**15**)*

**Scheme 3:**
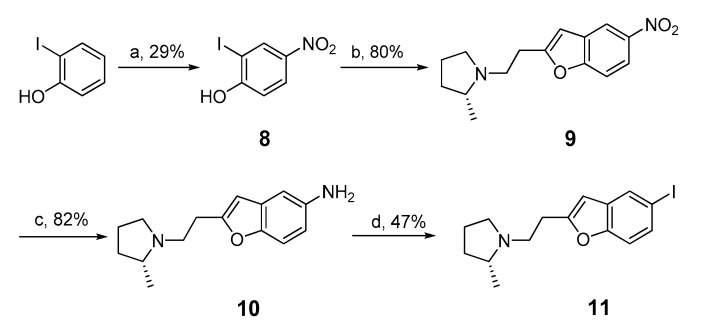
Synthesis of iodo-precursor **11**. *Conditions*: (**a**) HNO_3_; (**b**) (*R*)-1-(but-3-ynyl)-2-methylpyrrolidine, Pd(PPh_3_)_2_Cl_2_, CuI, DMF; (**c**) Pd/C, HCOOK; (**d**) NaNO_2_, KI.

The common (*R*)-1-(2-(5-benzofuran-2-yl)ethyl)-2-methylpyrrolidine structural feature in ligands **1**–**4** led us to synthesize a single iodo-precursor **11** in four steps from commercially available 2-iodophenol (Scheme 3). The 2-Iodophenol was first converted into 2-iodo-4-nitrophenol (**8**) by reaction with nitric acid [21] in moderate but useful 29% yield. Sonogashira cross-coupling [22] of **8** with (*R*)-1-(but-3-ynyl)-2-methylpyrrolidine gave the nitro derivative **9** in 80% yield. Compound **9** was reduced to the corresponding amine **10** in 82% yield with palladium on carbon in a solution of potassium formate. Iodination in the presence of sodium nitrite and potassium iodide [23] afforded iodoarene **11** in moderate yield (47%).

Trimethyl(4-nitrophenyl)stannane (**12**) was obtained in 90% yield by coupling *p*-iodonitrobenzene with hexamethylditin in the presence of Pd(MeCN_2_)Cl_2_, as previously reported [24]. The three other stannyl derivatives (**13**–**15**) were prepared with literature methods [25,26] by reaction of the appropriate Grignard reagent with trimethyltin bromide.

### 2.3. Radiosynthesis

**Scheme 4:**
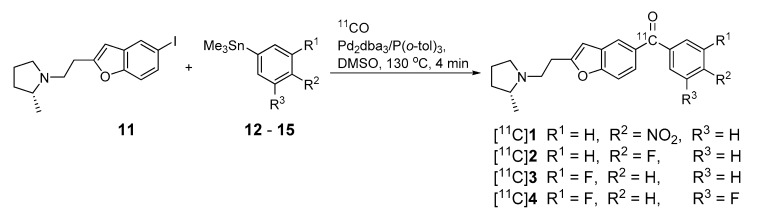
Radiosyntheses of [^11^C]**1**–**4** with Pd(0)-mediated ^11^C-carbonylative coupling of an iodoarene with aryltrimethylstannanes.

Initial labeling experiments with **11** and **12** using Pd(0) and xantphos in DMF or THF did not produce the target aryl ketone [^11^C]**1**, but yielded the corresponding [^11^C]acid by-product. The ^11^C-carbonylation reaction succeeded when DMSO was used as solvent and P(*o*-tol)_3_ as palladium ligand (Scheme 4). Subsequently, [^11^C]**1**–**4** were prepared according to a standard procedure using Pd(0) catalyzed ^11^C-carbonylation with iodoarene **11** and an appropriately substituted arylstannyl reagent (**12**–**15**) in DMSO.

When labeling was performed in the presence of air and moisture, we observed that more [^11^C]acid by-product was generated and lower yields of target [^11^C]aryl ketones were obtained. Therefore, all reagents were handled in a dry glove box under nitrogen gas. The Pd(0) reagent was instantly generated when Pd_2_(dba)_3_ and P(*o*-tol)_3_ were dissolved in anhydrous DMSO (80 µL). The palladium mixture was then added to the iodo-precursor in a capped polypropylene vial (250 µL) and kept in the glove box until 5–10 min before the beginning of radiosynthesis. Three min before the start of radiosynthesis, the tin reagent was added to the reaction mixture outside the glove box while the vial cap remained on and the final mixture in DMSO was uploaded to the reagent loop of the Synthia radiosynthesis platform. Cyclotron-produced [^11^C]CO_2_ was converted into [^11^C]CO in 75% yield through one pass over molybdenum wires at 875 °C with helium carrier gas flow at 10 mL/min. The cryogenically concentrated [^11^C]CO plus the reaction mixture in DMSO were compressed into the autoclave and sealed. The optimized temperature for ^11^C-carbonylation was 130 °C, and the reaction time 4 min.

The radioactive product was purified with semi-preparative scale reverse phase HPLC. After removal of solvent, the residue was reconstituted in a saline solution (10 mL) containing 10% (*v*/*v*) EtOH and ascorbic acid (0.5 mg), and filtered through a 0.2 µm sterile filter before being released for an imaging experiment. The addition of ascorbic acid helped to reduce radiolysis or decomposition of the radioligands.

Key radiosynthesis parameters are summarized in Table 2. No significant reactivity difference was observed between the four stannyl compounds. Overall decay-corrected yields of radiotracers for clinical use were in the range of 5–9% for each new radioligand whether substituted in *meta* or *para* position and whether bearing an electron-withdrawing or an electron-donating group. For the HPLC purification, we observed that a small change in pH of the aqueous mobile phase caused a shift in the product peak retention time. Therefore, it was good practice to use freshly prepared aqueous NH_4_OH solution. Incidentally, the lower molar activity of [^11^C]**2** coincided with the replacement of a new molecular sieves 13X column. It is possible that due to less rigorous heating activation in order to prolong its life, residual CO_2_ had been retained inside the molecular sieve pores and then released along with [^11^C]CO_2_. However, the molar activity obtained at the end of synthesis was still adequate for brain receptor imaging purposes. Molar activity data for the other three labeled compounds were consistently high.

## 3. Materials and Methods

Reference compounds **1**–**3** were prepared as reported previously [18] using the appropriately substituted benzophenones obtained from Combi-Blocks (San Diego, CA, USA). All other chemicals and solvents were purchased from Sigma-Aldrich (Milwaukee, WI, USA) or Alfa Aesar (Ward Hill, MA, USA) and used without further purification unless otherwise indicated.

The ^1^H- (400 MHz), ^19^F- (376.5 MHz) and ^13^C-NMR (100 MHz) spectra of all compounds were acquired on an Advance (Bruker) spectrometer using the chemical shifts of residual deuterated solvent as internal standard; chemical shifts (δ) for the proton and carbon resonance are reported in parts per million (ppm) downfield from TMS (δ = 0). Thin layer chromatography (TLC) was performed with silica gel layers (type 60 F254; EM Science) and compounds visualized under ultraviolet (UV) light. Mass spectra were acquired with a PolarisQ GC-MS instrument (Thermo Finnigan, San Jose, CA, USA) equipped with a capillary RTX-5ms column (30 m × 0.25 mm; flow rate, 1 mL/min; carrier gas, He). LC-MS was performed on a LCQ Deca instrument (Thermo Fisher Scientific Corp.; Waltham, MA, USA) equipped with a reversed-phase HPLC column (Luna C18, 3 μm, 50 mm × 2 mm; Phenomenex, Torrance, CA, USA), eluted at 200 μL/min with a mixture of A (H_2_O:MeOH:AcOH, 90:10:0.5 by vol.) and B (MeOH:AcOH, 100:0.5 *v*/*v*), initially composed of 20% B and linearly reaching 80% B in 3 min. Melting points were measured with a Digital SMP20 (Stuart) melting point apparatus and are uncorrected. Yields are reported for spectroscopically (^1^H-NMR) or chromatographically pure materials. HRMS data were acquired at the Mass Spectrometry Laboratory, University of Illinois at Urbana-Champaign (Urbana, IL, USA) under electron ionization conditions using a double-focusing high-resolution mass spectrometer (Micromass, Waters, Milford, MA, USA). All HRMS are provided for ESI+ (M^+^ + 1) except for compound **8**, obtained in EI+ (M).

Radioactive products were separated with HPLC on an apparatus comprising a solvent module (System Gold 126; Beckman Coulter, Fullerton, CA, USA) coupled with an absorbance detector operating at 254 nm (Model 166; Beckman Coulter) and a sodium iodide radioactivity detector (Bioscan, Washington, DC, USA). The HPLC apparatus for radioligand analysis comprised a solvent module (System Gold 126; Beckman Coulter) coupled with an absorbance detector operating at 254 nm (Model 168; Beckman Coulter) and a radioactivity detector (PMT, Flow-count; Bioscan). Radioactivity was measured with a calibrated dose ionization chamber (Atomlab 300; Biodex Medical Systems Inc., Shirley, NY, USA). All radioactivity measurements were corrected for background and were decay-corrected.

*(3,5-Difluorophenyl)(4-methoxyphenyl)methanone* (**5**). Scandium triflate (0.12 g, 0.25 mmol) was added to a solution of anisole (0.27 g, 0.271 mL, 2.5 mmol) and 3,5-difluorobenzoyl chloride (0.44 g, 2.5 mmol) in nitroethane (5 mL). The resultant dark-purple solution was stirred at 60 °C for 5 days at which point TLC showed no remaining starting material. The solution was quenched with saturated NaHCO_3_ (10 mL) and extracted thrice with DCM. The solvent from the combined organic layers was dried over MgSO_4_, evaporated under vacuum, and the residue was purified on silica gel (hexanes:EtOAc, 90:10) to give **5** (0.36 g, 59%) as white crystals. m.p.: 90–92 °C. ^1^H-NMR (CDCl_3_) δ 7.81 (m, 2H), 7.26 (m, 2H), 7.00 (m, 3H), 3.90 (s, 3H). ^13^C-NMR (CDCl_3_) δ 192.64, 163.80, 162.64 (dd, *J*_1_ = 251.0 Hz, *J*_2_ = 11.8 Hz), 141.35 (t, *J*_1_ = 251.0 Hz, *J*_2_ = 11.8 Hz), 132.55, 128.98, 113.87, 112.56 (dd, *J*_1_ = 25.2 Hz, *J*_2_ = 11.2 Hz), 107.13 (t, *J* = 25.3 Hz), 55.58. ^19^F-NMR (CDCl_3_) δ −108.41. HRMS ESI: calc’d 249.0722 for C_14_H_11_O_2_F_2_; found 249.0735.

*(3,5-Difluorophenyl)(4-hydroxyphenyl)methanone* (**6**). A solution of compound **5** (0.240 g, 0.97 mmol) in DCM (5 mL) was placed in a round-bottom flask under argon and cooled to −78 °C. A solution of boron tribromide (2.9 mL, 2.9 mmol, 0.1 M in DCM) was added dropwise at −78 °C and the reaction mixture was allowed to warm to room temperature (RT) and stirred until no starting material was observed with TLC. The reaction mixture was cooled to 0 °C in an ice-bath and quenched by slow addition of water (1 mL). After stirring at 0 °C for 5 min, more water was added and the layers were separated. The aqueous layer was extracted twice with DCM (10 mL) and the combined organic layers were dried over MgSO_4_ and evaporated to dryness. Chromatography on silica gel (pentane:Et_2_O, 60:40) gave **6** (0.202 g, 89%) as a beige powder. m.p.: 150–151 °C. ^1^H-NMR (MeOD) δ 7.73 (m, 2H), 7.26 (m, 3H), 6.90 (m, 2H). ^13^C-NMR (MeOD) δ 194.30, 164.28, 164.14 (dd, *J*_1_ = 249.6 Hz, *J*_2_ = 12.2 Hz), 143.31 (t, *J* = 7.8 Hz), 134.00, 128.82, 116.44, 113.36 (dd, *J*_1_ = 26.4 Hz, *J*_2_ = 11.7 Hz), 107.84 (t, *J* = 25.9 Hz). ^19^F-NMR (CDCl_3_) δ −110.46. HRMS calc’d 235.0571 for C_13_H_9_O_2_F_2_; found 235.0568.

*(3,5-Difluorophenyl)(4-hydroxy-iodophenyl)methanone* (**7**). **6** (0.92 g, 3.93 mmol) was dissolved in 28–30% aq. ammonium hydroxide (60 mL) for 20 min at RT. An aqueous solution (15 mL) of potassium iodide (3.26 g, 19.7 mmol) and iodine (1.00 g, 3.93 mmol) was added and the mixture was stirred for 10 min. A solution of 6 M HCl was then added until neutral pH was reached and then the resultant solution was extracted thrice with AcOEt (40 mL). The organic layers were combined, washed with water (40 mL), brine (40 mL), dried over MgSO_4_ and then evaporated to dryness. Chromatography on silica gel eluting with DCM gave **7** (0.956 g, 67%) as an off-white solid. m.p.: 208–210 °C. ^1^H-NMR (DMSO-*d*_6_) δ 11.50 (br s, 1H), 8.11 (d, *J* = 2.1 Hz, 1H), 7.66 (dd, *J*_1_ = 8.5 Hz, *J*_2_ = 2.1 Hz, 2H), 7.56 (m, 1H), 7.35 (d, *J* = 5.56 Hz, 2H), 7.01 (d, *J* = 8.5 Hz, 1H). ^13^C-NMR (DMSO-*d*_6_) δ 190.56, 162.01 (dd, *J*_1_ = 248.7 Hz, *J*_2_ = 12.4 Hz), 161.56, 141.02, 140.97, 132.57, 128.69, 114.48, 112.23 (dd, *J*_1_ = 26.4 Hz, *J*_2_ = 11.4 Hz), 107.27 (t, *J* = 25.8 Hz), 85.04. ^19^F-NMR (DMSO-*d*_6_) δ −108.37. HRMS ESI: calc’d. 360.9537 for C_13_H_8_O_2_F_2_I; found 360.9540.

*(R)-(3,5-Difluorophenyl)(2-(2-(2-methylpyrrolidin-1-yl)ethyl)benzofuran-5-yl)methanone* (**4**). (*R*)-1-(But-3-ynyl)-2-methylpyrrolidine (0.1 M in acetonitrile) was first prepared by reaction of (*R*)-2-methylpyrrolidine (0.60 g, 7.0 mmol), potassium carbonate (2.03 g, 14.7 mmol) and 3-butynyl 4-toluenesulfonate (1.57 g, 7.0 mmol) in MeCN (60 mL). The mixture was stirred at 23 °C for 1 h and then heated to 50 °C for 24 h. The mixture was allowed to cool to RT. The precipitated salts were filtered off and the filter cake was washed with small amounts of MeCN. The filtrate was diluted to a total volume of 70 mL and used as a solution of 0.1 M reagent.

A round-bottom flask under nitrogen was charged with **7** (0.47 g, 1.3 mmol), Pd(PPh_3_)_2_Cl_2_ (0.04 g, 0.06 mmol, 3.5%) and CuI (0.012 g, 0.07 mmol, 5%) in DMF (3 mL). A mixture of triethylamine (0.26 g, 2.6 mmol) in DMF (2 mL) and (*R*)-1-(but-3-ynyl)-2-methylpyrrolidine (17 mL, 1.7 mmol from the stock solution) was then introduced into the flask. The mixture was stirred at RT for 30 min and then heated to 60 °C for 12 h. The reaction mixture was cooled, diluted with water and extracted three times with AcOEt. The combined organic layers were washed thrice with water, dried over MgSO_4_, and filtered. Solvent was removed under vacuum, and the crude residue was purified with silica gel (DCM:MeOH:NH_4_OH, 97:2.9:0.1) to give **4** (0.289 g, 61%) as a heavy oil. ^1^H-NMR (DMSO-*d*_6_) δ 7.82 (d, *J* = 1.4 Hz, 1H), 7.58 (dd, *J*_1_ = 8.5 Hz, *J*_2_ = 1.8 Hz, 1H), 7.41 (d, *J* = 8.6 Hz, 1H), 7.14 (m, 3H), 6.56 (d, *J* = 0.6 Hz, 1H), 3.17 (m, 2H), 2.95 (m, 2H), 2.48 3.17 (m, 2H), 2.26 (qd, *J* = 8.9 Hz, 1H), 1.91 (m, 1H), 1.70 (m, 2H), 1.36 (m, 1H), 1.07 (d, *J* = 6.2 Hz, 3H). ^13^C-NMR (DMSO-*d*_6_) δ 195.03, 165.36 (dd, *J*_1_ = 249.8 Hz, *J*_2_ = 12.1 Hz), 160.43, 158.81, 142.85 (t, *J* = 7.9 Hz), 132.67, 130.42, 127.13, 124.76, 113.71 (dd, *J*_1_ = 26.6 Hz, *J*_2_ = 11.5 Hz), 112.01, 108.27 (t, *J* = 25.3 Hz), 104.59, 62.11, 54.69, 52.75, 33.34, 28.19, 22.40, 18.35. ^19^F-NMR (DMSO-*d*_6_) δ −108.37. HRMS ESI: calc’d. 370.1613 for C_22_H_22_F_2_NO_2_; found 370.1610.

*2-Iodo-4-nitrophenol* (**8**). A 70% nitric acid solution (0.70 mL, 10.9 mmol) was slowly added to a solution of 2-iodophenol (2.0 g, 9.1 mmol) in DCM (20 mL). The reaction was stirred for 6 h at RT, then diluted with water and extracted thrice with DCM. The solvent from the combined organic layers was evaporated off, and the compound was purified on silica gel (hexanes:EtOAc, 95:5 to 80:20) to give **8** (0.705 g, 29%) as a yellow solid. m.p.: 85–86 °C. (lit. m.p.: 84–86 °C [27]). ^1^H-NMR (DMSO-*d*_6_) δ 11.96 (br s, 1H), 8.51 (d, *J* = 2.76 Hz, 1H), 8.15 (dd, *J*_1_ = 8.98 Hz, *J*_2_ = 2.78 Hz, 1H), 7.02 (d, *J* = 9.00 Hz, 1H). ^13^C-NMR (DMSO-*d*_6_) δ 161.86, 138.88, 133.24, 124.50, 113.03, 83.13. HRMS EI+ calc’d 264.92362 for C_6_H_4_O_3_NI; found 264.92486.

*(R)-2-Methyl-1-(2-(5-nitrobenzofuran-2-yl)ethyl)pyrrolidine* (**9**). A round-bottom flask was charged with **8** (0.66 g, 2.5 mmol), Pd(PPh_3_)_2_Cl_2_ (0.06 g, 0.09 mmol, 3.5%) and CuI (0.02 g, 0.13 mmol, 5%) in DMF (5 mL). A mixture of triethylamine (0.51 g, 5.0 mmol) in DMF (2 mL) and (*R*)-1-(but-3-ynyl)-2-methylpyrrolidine (35 mL, 3.5 mmol) was then introduced into the flask. The mixture was stirred at RT for 30 min and then heated to 60 °C for 18 h. The reaction mixture was cooled, diluted with water and extracted thrice with DCM. The combined organic layers were washed thrice with 0.5 M NaOH and thrice with water, dried over MgSO_4_, and filtered. Solvent was removed under vacuum, and the crude residue was purified with silica gel (DCM:MeOH:NH_4_OH, 97:2.7:0.3) to give **9** (0.547 g, 80%) as a heavy brown oil. ^1^H-NMR (CDCl_3_) δ 8.40 (d, *J* = 2.36 Hz, 1H), 8.14 (dd, *J*_1_ = 8.98 Hz, *J*_2_ = 2.38 Hz, 1H), 7.47 (d, *J* = 8.88 Hz, 1H), 6.60 (d, *J* = 0.92 Hz, 1H), 3.25 (m, 2H), 3.05 (t, *J* = 7.72 Hz, 2H), 2.53 (m, 1H), 2.43 (m, 1H), 2.24 (q, *J* = 8.79 Hz, 1H), 1.95 (m, 1H), 1.77 (m, 2H), 1.47 (m, 1H), 1.15 (d, *J* = 6.08 Hz, 3H). ^13^C-NMR (CDCl_3_) δ 161.42, 157.49, 143.97, 129.32, 119.28, 116.73, 110.95, 103.34, 60.12, 53.79, 51.52, 32.67, 28.07, 21.70, 18.88. HRMS ESI: calc’d 275.1396 for C_15_H_19_O_3_N_2_; found 275.1394.

*(R)-2-(2-(2-Methylpyrrolidin-1-yl)ethyl)benzofuran-5-amine* (**10**). Pd/C (10%, 0.30 g) and potassium formate (1.24 g, 15.1 mmol) [28] were added to a solution of **9** (0.473 g, 1.72 mmol) in MeOH (10 mL), and the mixture refluxed at 70 °C for 2 h. The reaction mixture was allowed to cool to RT, and then filtered over Celite. Concentrated HCl (12 M) was added dropwise to the filtrate until no more effervescence was observed. The mixture was filtered again. The filtrate was concentrated under vacuum, and the resultant residue was partitioned between EtOAc and aq. KHCO_3_ (1.0 M). The organic layer was evaporated under vacuum to give a crude product. The crude compound was purified by partitioning between EtOAc and HCl (aq. 1 M), and the pH of the aqueous layer was then adjusted to 10 with NaOH (aq. 2 M). The resulting liquid was extracted thrice with EtOAc. The combined organic layers were dried over MgSO_4_ and filtered. Removal of solvent under vacuum gave **10** (0.344 g, 82%) as a brown semi-solid. ^1^H-NMR (CD_3_CN) δ 7.12 (d, *J* = 8.56 Hz, 1H), 6.70 (dd, *J*_1_ = 2.38 Hz, *J*_2_ = 0.54 Hz, 1H), 6.54 (dd, *J*_1_ = 8.62 Hz, *J*_2_ = 2.34 Hz, 1H), 6.32 (d, *J* = 0.96 Hz, 1H), 3.93 (br s, NH_2_), 3.15 (m, 2H), 2.87 (m, 2H), 2.35 (m, 2H), 2.14 (m, 1H), 1.90 (m, 1H), 1.67 (m, 2H), 1.32 (m, 1H), 1.05 (d, *J* = 6.04, 3H). ^13^C-NMR (CD_3_CN) δ 158.13, 148.05, 143.26, 129.47, 111.41, 110.14, 104.16, 101.64, 59.35, 52.99, 51.27, 32.26, 27.45, 21.13, 18.06. HRMS ESI: calc’d 245.1654 for C_15_H_21_N_2_O; found 245.1650.

*(R)-1-(2-(5-Iodobenzofuran-2-yl)ethyl)-2-methylpyrrolidine* (**11**). *p*-Toluenesulfonic acid (0.70 g, 3.7 mmol) was added to a solution of **10** (0.30 g, 1.2 mmol) in MeCN (6 mL). The resulting suspension was cooled to 5 °C. A solution of sodium nitrite (0.17 g, 2.5 mmol) and potassium iodide (0.51 g, 3.1 mmol) in water was added dropwise, releasing N_2_ gas. The reaction mixture was stirred for 10 min, and then gradually warmed to RT over 50 min. The mixture was diluted with water and pH adjusted to 10 by NaHCO_3_ (aq. 1.0 M). Liquid was extracted thrice with EtOAc. The combined organic layers were dried over MgSO_4_, and solvent was evaporated under reduced pressure. Crude product was purified on silica gel (DCM:MeOH:NH_4_OH, 95:4.5:0.5) to give **11** (0.204 g, 47%) as a brown semi-solid. ^1^H-NMR (CD_3_CN) δ 7.87 (d, *J* = 1.80 Hz, 1H), 7.51 (dd, *J*_1_ = 8.56 Hz, *J*_2_ = 1.84 Hz, 1H), 7.24 (dd, *J*_1_ = 8.58 Hz, *J*_2_ = 0.42 Hz, 1H), 6.51 (d, *J* = 0.96 Hz, 1H), 3.19 (m, 2H), 2.95 (m, 2H), 2.44 (m, 2H), 2.20 (m, 1H), 1.91 (m, 1H), 1.68 (m, 2H), 1.34 (m, 1H), 1.07 (d, *J* = 7.40 Hz, 3H). ^13^C-NMR (CD_3_CN) δ 159.21, 153.56, 131.56, 131.42, 128.83, 112.45, 101.46, 85.19, 59.60, 52.93, 50.90, 32.14, 27.06, 21.10, 17.83. HRMS calc’d 356.0511 for C_15_H_19_NOI; found 356.0504.

*Trimethyl(4-nitrophenyl)stannane* (**12**). 1-Iodo-4-nitrobenzene (0.249 g, 1.00 mmol) and Sn_2_Me_6_ (0.426 g, 1.30 mmol) were added to DMF (5 mL). Pd(CH_3_CN)_2_Cl_2_ (0.006 g, 0.03 mmol) was added, and the reaction was stirred at RT for 10 min. The reaction mixture was diluted with water and extracted thrice with diethyl ether. The combined organic layers were washed thrice with water, dried over MgSO_4_, and filtered. The solvent from the filtrate was evaporated under reduced pressure, and the crude residue was purified on a silica gel plug column (hexane: diethyl ether, 90:10) to give **9** (0.258 g, 90%) as a light yellow solid. m.p.: 49–51 °C (lit. m.p.: 47–48 °C [29]).

*Radiosynthesis and purification.* Radiosynthesis was performed with a modified Synthia [^11^C]CO module [30]. In a typical procedure, iodoarene **11** (1.4 mg, 3.9 µmol), 4-fluoroaryltrimethylstannane (**13**, 1.4 mg, 5.3 µmol), tris(dibenzylidene-acetone)dipalladium(II) (0.4 mg, 0.45 µmol), and P(*o*-tol)_3_ (0.9 mg, 2.9 µmol) were mixed with DMSO (80 µL) and loaded into the autoclave of the apparatus. Each compound and reagent was measured and mixed under nitrogen gas protection in a glove box. [^11^C]CO, generated by a single pass of cyclotron-produced [^11^C]CO_2_ over heated (875 °C) Mo wires, was cryogenically concentrated and then directed into the autoclave containing reagent mixture. The autoclave was sealed and heated at 130 °C for 4 min. The reaction mixture was then flushed out with THF (0.7 mL) into a collection vial (5-mL), diluted with water (3 mL) and injected onto a Luna C18 column (250 × 10 mm) eluted using semi-prep HPLC method A or B for purification of the radioligand (See Figures S1–S4 for examples of HPLC chromatograms). Upon removal of solvent, the final dose-for-injection was formulated in saline-10% *v*/*v* EtOH containing ascorbic acid (0.5 mg) for intravenous injection. Radioligand purities and molar activities were measured with analytical reverse phase HPLC, and radioligand identities were confirmed by LC-MS of associated carrier and co-elution with the non-radioactive standards.

*Semi-prep HPLC method A*. The reaction mixture was eluted on Luna C18 column (5 µm, 250 × 10 mm). The mobile phase consisted of aqueous NH_4_OH (A, 1 mM, pH = 8.5) and MeCN (B) with B initially at 40% (*v*/*v*) for 2 min and increased to 88% in 2 min at a flow rate of 6 mL/min. The eluate was monitored for radioactivity and UV absorbance (254 nm).

*Semi-prep HPLC method B*. The reaction mixture was eluted on Luna C18 column (5 µm, 250 × 10 mm). The mobile phase consisted of aqueous NH_4_OH (A, 5 mM, pH = 9.0) and MeCN (B) with B initially at 40% (*v*/*v*) for 2 min and increased to 82% in 2 min at a flow rate of 6 mL/min. The eluate was monitored for radioactivity and UV absorbance (254 nm).

*Analytical HPLC method for QC and SA determination*. The column was a Luna C18 column (10 µ, 250 × 4.0 mm). For [^11^C]**1**, the mobile phase was an isocratic mixture of MeCN (85%) and aq. NH_4_OH (1 mM, pH = 8.5, 15%) eluted at 2 mL/min. For [^11^C]**2**–**4**, the mobile phase consisted aq. NH_4_OH (A, 1 mM, pH = 8.5) and MeCN (B) with B initially at 45% (*v*/*v*) for 1 min and increased to 91% in 4 min at a flow rate of 2.75 mL/min. The eluate was monitored for radioactivity and UV absorbance (254 nm).

## 4. Conclusions

Pd(0)-mediated ^11^C-carbonylative coupling of iodoarenes with aryltrimethylstannanes proved to be an effective method to synthesize ^11^C-labeled aryl ketones [^11^C]**1**–**4** as prospective high-affinity and selective H3R radioligands with adequate yield, chemical purity, radiochemical purity, and molar activity.

## Figures and Tables

**Table 1:** *K*_i_ values determined from in vitro competitive binding assays and calculated LogD for **1**–**4**.

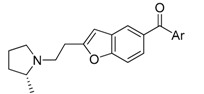						
Ligand	Ar	Affinity (*K*_i_, nM)				cLogD
		H_3_ (lit) ^a^	H_3_^b^	H_2_^c^	H_1_^c^	
**1**	4-NO_2-_C_6_H_4_	-	2.1	706	1030	2.69
**2**	4-F_-_C_6_H_4_	0.27	4.2	923	7055	2.90
**3**	3-F_-_C_6_H_4_	0.10	4.9	1368	>10,000	2.79
**4**	3,5-di-F_-_C_6_H_3_	0.09	2.9	630	>10,000	2.75

^a^ Assayed by displacement of [^3^H]α-methylhistamine from cell membranes isolated from C6 cells expressing cloned human H_3_ receptors [18]; ^b^ Assays performed by displacing [^3^H]α-methylhistamine from guinea pig cells expressing H_3_ receptors at the NIMH’s Psychoactive Drug Screening Program (PDSP); ^c^ Assays performed by displacing [^3^H]tiotidine for H2R, and [^3^H]pyrilamine for H1R from human recombinant receptors expressed in HEK293T cells at PDSP.

**Table 2:** Summary of key radiosynthesis parameters.

Radioligand	Yields ^a^ (%)	Molar activity at EOS ^b^ (GBq/µmol)	Prep-HPLC Method	*t*_R_ (min)
[^11^C]**1**	4.9 (n = 10)	293 ± 84	A	13.1
[^11^C]**2**	6.8 (n = 5)	115 ± 45	A	14.4
[^11^C]**3**	6.1 (n = 4)	420 ± 60	B	14.3
[^11^C]**4**	8.6 (n = 3)	398 ± 141	B	15.1

^a^ Based on the formulated dose from cyclotron produced [^11^C]CO_2_; ^b^ EOS = end of synthesis.

## Supplementary Materials

The supplementary materials are available online. Examples of radio-HPLC chromatograms (A) Semi-prep, (B) QC for [^11^C]**1**–**4**.
