# Supplementary material for: 11C-Labeling of Aryl Ketones as Candidate Histamine Subtype-3 Receptor PET Radioligands through Pd(0)-Mediated 11C-Carbonylative Coupling
Source: Molecules. 2017 May 12;22(5):792. doi: 10.3390/molecules22050792 (PMC5530730; doi:10.3390/molecules22050792)
Supplement: Supplementary file 1 [file molecules-22-00792-s001.pdf]

Supplementary Material

^{11}C-Labeling of aryl ketones as candidate histamine subtype-3 receptor PET radioligands through Pd(0)-mediated ^{11}C-carbonylative coupling

Fabrice G. Siméon, William J. Culligan, Shuiyu Lu and Victor W. Pike*

Molecular Imaging Branch, National Institute of Mental Health, National Institutes of Health, Bethesda, Maryland, 20892-1003, USA

Figure S1. An example of Semi-prep (A) and QC (B) radio-HPLC chromatograms for [^{11}C]**1**
Figure S2. An example of Semi-prep (A) and QC (B) radio-HPLC chromatograms for [^{11}C]**2**
Figure S3. An example of Semi-prep (A) and QC (B) radio-HPLC chromatograms for [^{11}C]**3**
Figure S4. An example of Semi-prep (A) and QC (B) radio-HPLC chromatograms for [^{11}C]**4**

(A) [^{11}C]**1** semi-prep HPLC

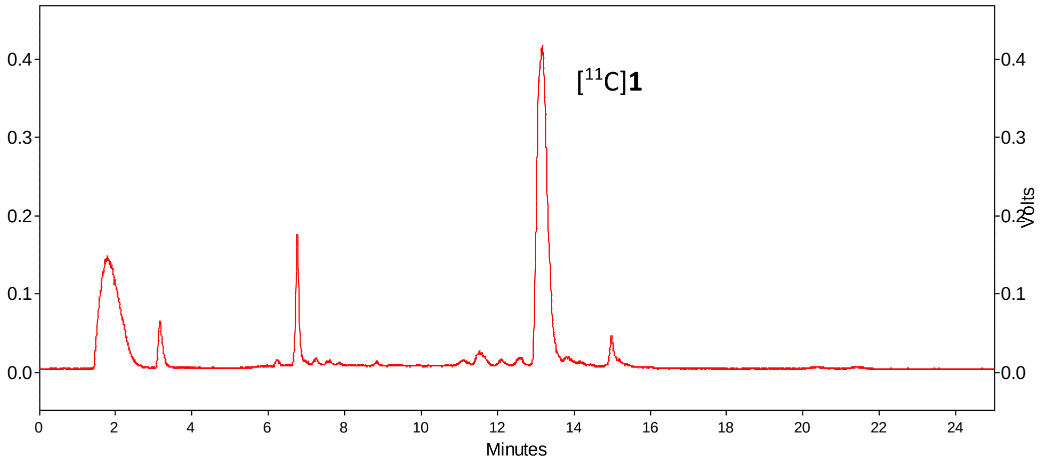

(B) [^{11}C]**1** QC HPLC

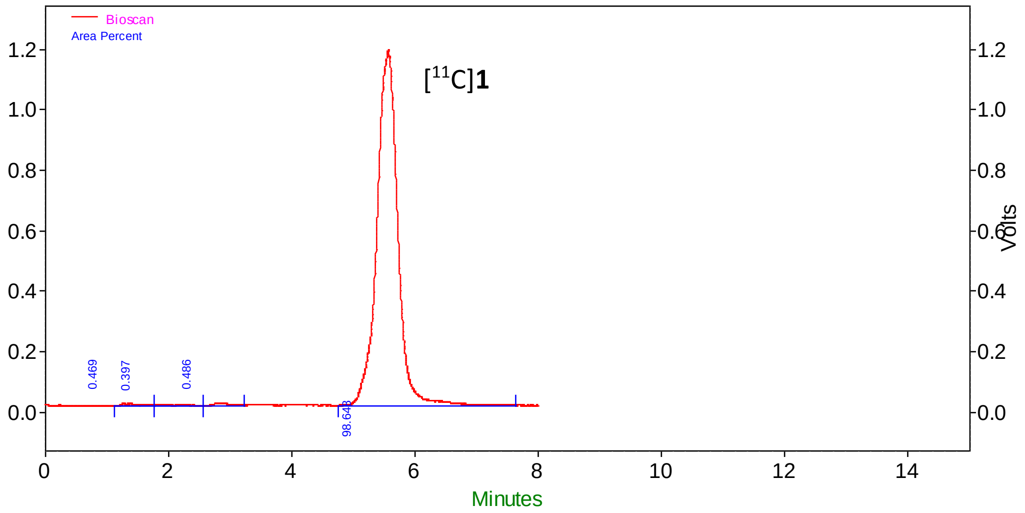

Figure S1. An example of Semi-prep (A) and QC (B) radio-HPLC chromatograms for [^{11}C]**1**.

(A) [^{11}C]**2** semi-prep HPLC

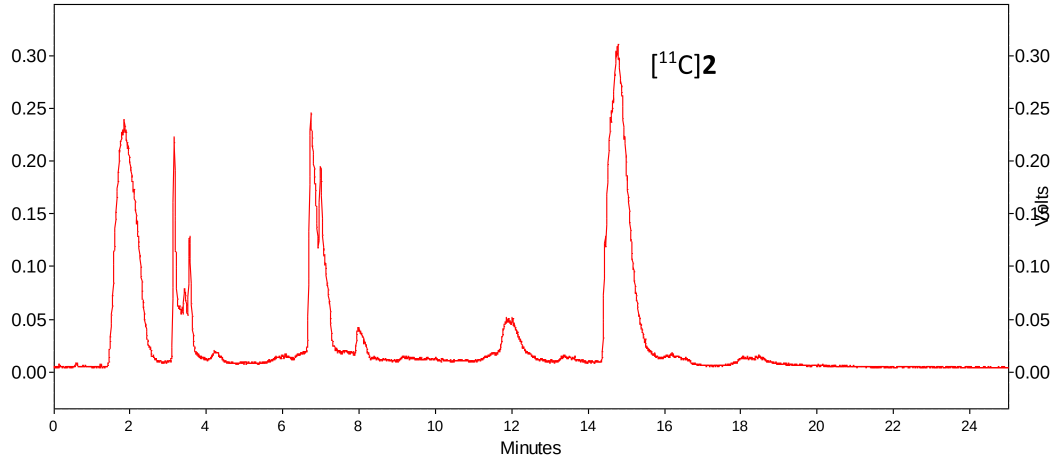

(B) [^{11}C]**2** QC HPLC

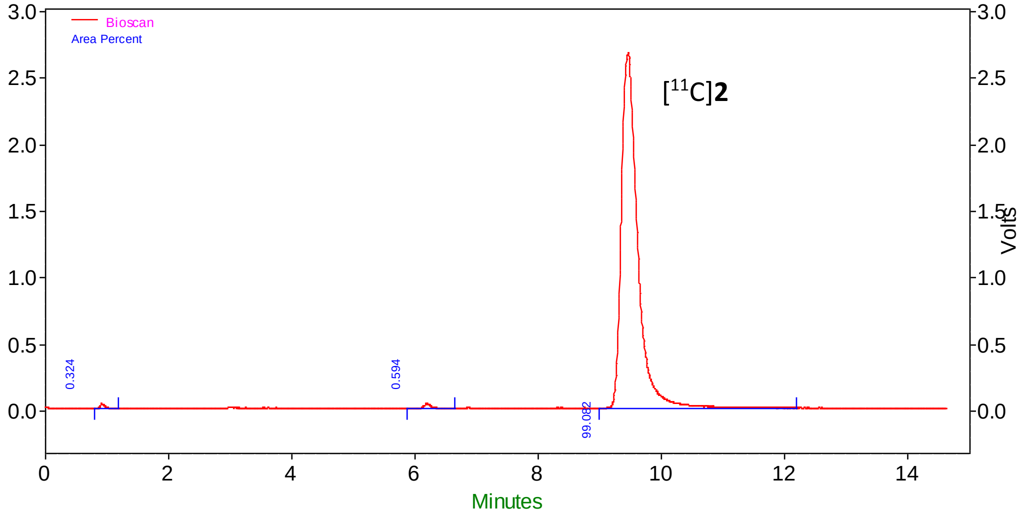

Figure S2. An example of Semi-prep (A) and QC (B) radio-HPLC chromatograms for [^{11}C]**2**.

(A) [^{11}C]**3** semi-prep HPLC

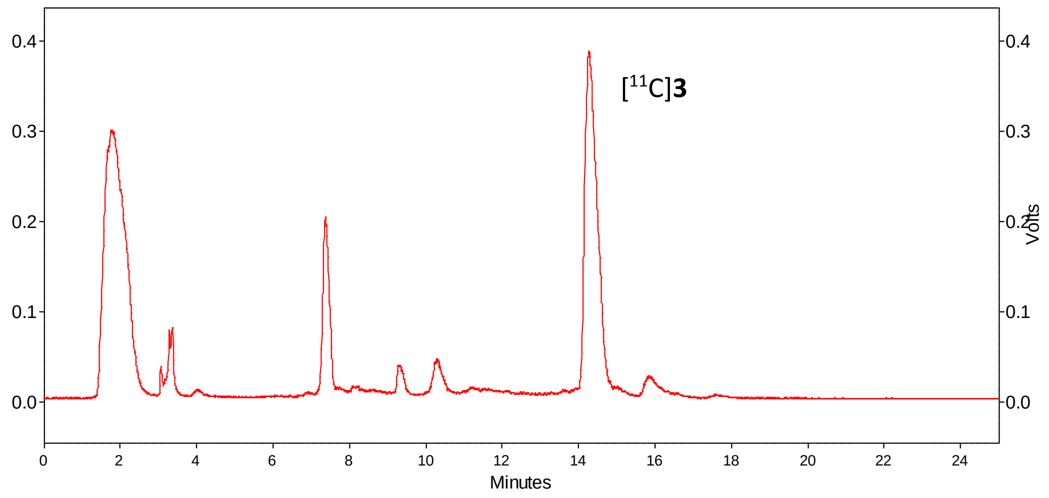

(B) [^{11}C]**3** QC HPLC

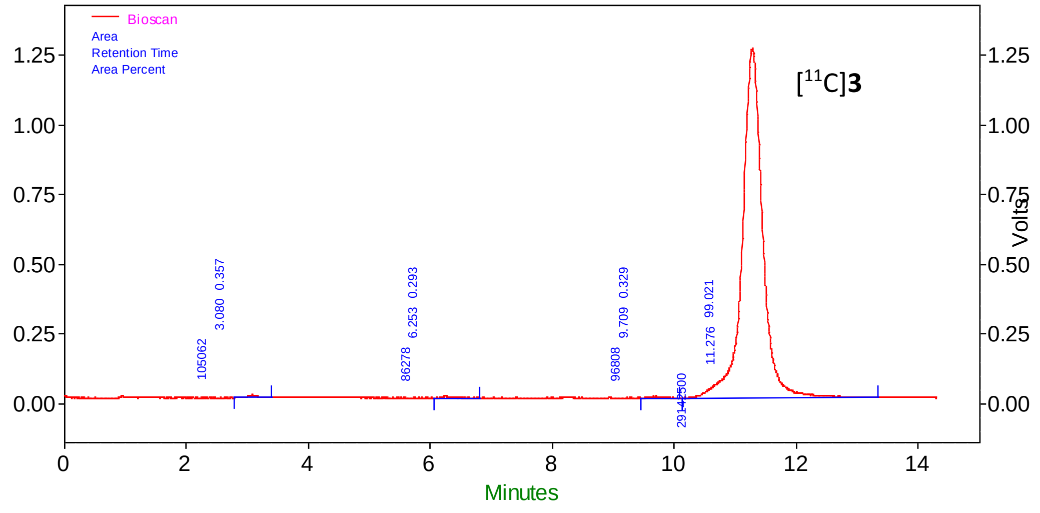

Figure S3. An example of Semi-prep (A) and QC (B) radio-HPLC chromatograms for [^{11}C]**3**.

(A) [^{11}C]**4** semi-prep HPLC

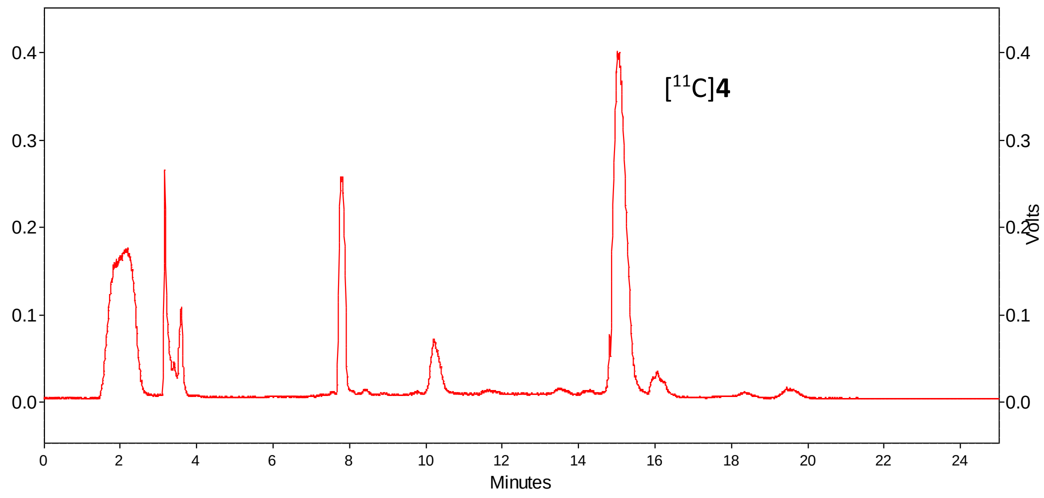

(B) [^{11}C]**4** QC HPLC

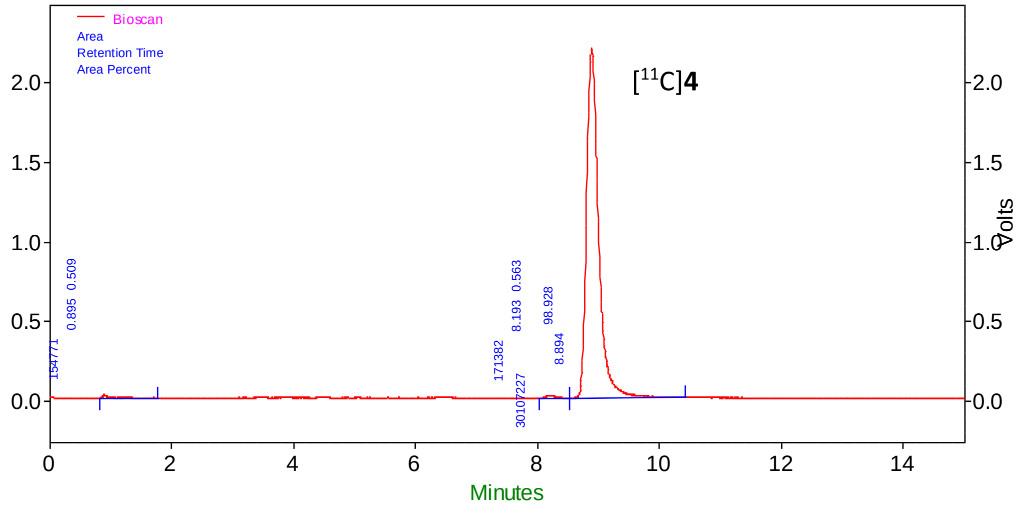

Figure S4. An example of Semi-prep (A) and QC (B) radio-HPLC chromatograms for [^{11}C]**4**.
